# Diagnosis value of aberrantly expressed microRNA profiles in lung squamous cell carcinoma: a study based on the Cancer Genome Atlas

**DOI:** 10.7717/peerj.4101

**Published:** 2017-11-30

**Authors:** Sheng Yang, Jing Sui, Geyu Liang

**Affiliations:** Key Laboratory of Environmental Medicine Engineering, Ministry of Education, School of Public Health, Southeast University, Nanjing, Jiangsu, China

**Keywords:** TCGA, MicroRNA, Diagnostic Biomarker, LUSC

## Abstract

**Background:**

Lung cancer is considered as one of the most frequent and deadly cancers with high mortality all around the world. It is critical to find new biomarkers for early diagnosis of lung cancer, especially lung squamous cell carcinoma (LUSC). The Cancer Genome Atlas (TCGA) is a database which provides both cancer and clinical information. This study is a comprehensive analysis of a novel diagnostic biomarker for LUSC, based on TCGA.

**Methods and Results:**

The present study investigated LUSC-specific key microRNAs (miRNAs) from large-scale samples in TCGA. According to exclusion criteria and inclusion criteria, the expression profiles of miRNAs with related clinical information of 332 LUSC patients were obtained. Most aberrantly expressed miRNAs were identified between tumor and normal samples. Forty-two LUSC-specific intersection miRNAs (fold change >2, *p* < 0.05) were obtained by an integrative computational method, among them six miRNAs were found to be aberrantly expressed concerning characteristics of patients (gender, lymphatic metastasis, patient outcome assessment) through Student *t*-test. Five miRNAs correlated with overall survival (log-rank *p* < 0.05) were obtained through the univariate Cox proportional hazards regression model and Mantel–Haenszel test. Then, five miRNAs were randomly selected to validate the expression in 47 LUSC patient tissues using quantitative real-time polymerase chain reaction. The results showed that the test findings were consistent with the TCGA findings. Also, the diagnostic value of the specific key miRNAs was determined by areas under receiver operating characteristic curves. Finally, 577 interaction mRNAs as the targets of 42 LUSC-specific intersection miRNAs were selected for further bioinformatics analysis.

**Conclusion:**

This study indicates that this novel microRNA expression signature may be a useful biomarker of the diagnosis for LUSC patients, based on bioinformatics analysis.

## Background

Lung cancer is one of the most lethal types of cancer, causing high mortality worldwide (Seifert, Schlanstedt-Jahn & Klug, 2015; Siegel, Miller & Jemal, 2016). In China, lung cancer occupies 17.09% of cancer cases with 24.35% mortality rate, ranking the most common and most lethal among all cancer types (Chen et al., 2016). The vast majority of lung cancers are classified as non-small cell lung cancer (NSCLC), which contains two major histologic subtypes: lung squamous cell carcinoma (LUSC) and lung adenocarcinoma (LUAD) (Torre, Siegel & Jemal, 2016). Advanced treatment strategies like radiotherapy, chemotherapy and surgical treatment have been developing rapidly, but the five-year overall survival of lung cancer remains poor (Wang et al., 2016). So, it is of great importance to seek methods for early detection, diagnosis, and treatment.

Because the genetic and epigenetic alterations between LUSC and LUAD are quite different (Mukhopadhyay & Katzenstein, 2011), it is significant to distinguish them for diagnosis and different treatments. So, increasing new biomarkers has afforded to improving the overall survival of lung adenocarcinoma patients (Chung et al., 2012; Pu et al., 2016; Wiedl et al., 2011). Unfortunately, effective biomarkers to diagnose LUSC patients for reducing recurrence and improving survival rate are still lacking. Therefore, altogether, it is important to find new biomarkers for prompt diagnosis and effective treatment options of LUSC.

MicroRNAs (miRNAs) are non-coding, single-stranded RNA molecules of approximately 18–25 nucleotides that can regulate the gene expression and protein-coding by binding to complementary sequences in 3′ or 5′ untranslated region (UTR) of target miRNAs (Bartel, 2004, 2009). Recently, numerous studies have reported that miRNAs can play important roles in the development and progression of various cancers by promoting the expression of oncogenes or by inhibiting suppressor genes (Liu et al., 2012; Luo et al., 2015; Nana-Sinkam & Croce, 2011; Suzuki et al., 2014; Wei et al., 2014), including lung cancers (Sadaf et al., 2012). This suggests that miRNAs may be potential new biomarkers for LUSC. However, most early studies identified lung cancer-related miRNAs from small sample sizes or only one or few key miRNAs (Chou et al., 2010; Remon et al., 2016), such as Huang, Yang & Cai (2015) analyzed 129 LUSC samples in The Cancer Genome Atlas (TCGA) and built the whole genome integrative network by the Bayesian method to find novel candidate key, such as the methylation of ARHGDIB and HOXD3, microRNA let-7a and miR-31, and the CNV of AGAP2. In the present study, data including miRNA expression profiles and detailed clinical information of LUSC were downloaded from TCGA database, a large-scale public data platform that is available to the public. It is a new method to predict and identify related miRNAs of LUSC to enhance the reliability and accuracy of the current research.

## Methods

### TCGA dataset and sample information

Up to February 12, 2017, a total of 504 LUSC samples with related information were obtained from the TCGA database. According to the inclusion criteria: (1) histologic diagnosis was LUSC; (2) data of samples were available on expression of genes and characteristics of patients. For the exclusion criteria: (1) first histologic diagnosis was not LUAD; (2) patients suffered from other malignant neoplasms except LUSC; and (3) overall survival more than five years. At last, this study embraced 332 eligible patients. Among these 332 LUSC, related information of LUSC tissue samples was from 293 patients and adjacent non-tumorous information were from 39 subjects. In addition, personal comprehensive sources including related clinical information and RNA expression data on LUSC patients were also obtained from the Data Coordinating Center. Among these 293 cases, 105 LUSC subjects had lymphatic metastases and 188 LUSC subjects had no lymphatic metastases. Moreover, according to the staging system of the Union for International Cancer Control (UICC), well or moderately differentiated LUSC (stage I–II) were 243 cases, and poorly differentiated LUSC (stage III–IV) were the remaining 50 cases. Because the data were provided by the TCGA, there was no need for approval of the Ethics Committee. This study fully met the guideline of the NIH TCGA human subject protection and data access policies.

In addition, 47 LUSC tissues samples were obtained from the Nanjing Chest Hospital of Southeast University. These samples including tumor tissues and adjacent non-tumor tissues were obtained immediately after surgical resection from patients who do not undergo preoperative radiotherapy or chemotherapy. These patients consented in advance and signed informed consent forms. Tissues were stored in RNA later (Ambion, Austin, TX, USA) at −80 °C until further use. This study was approved by the ethics committee of Zhongda Hospital Southeast University.

### Differential analysis of expressed miRNAs in LUSC samples

The LUSC RNA expression data (level 3) of patients were collected from the TCGA Data Portal. The TCGA database provided the normalized expression profile data of RNA sequencing including mRNAs and miRNAs by RNASeqV2 and Illumina HiSeq 2000 miRNA sequencing platforms (Illumina Inc., Hayward, CA, USA), respectively.

**Figure 1:**
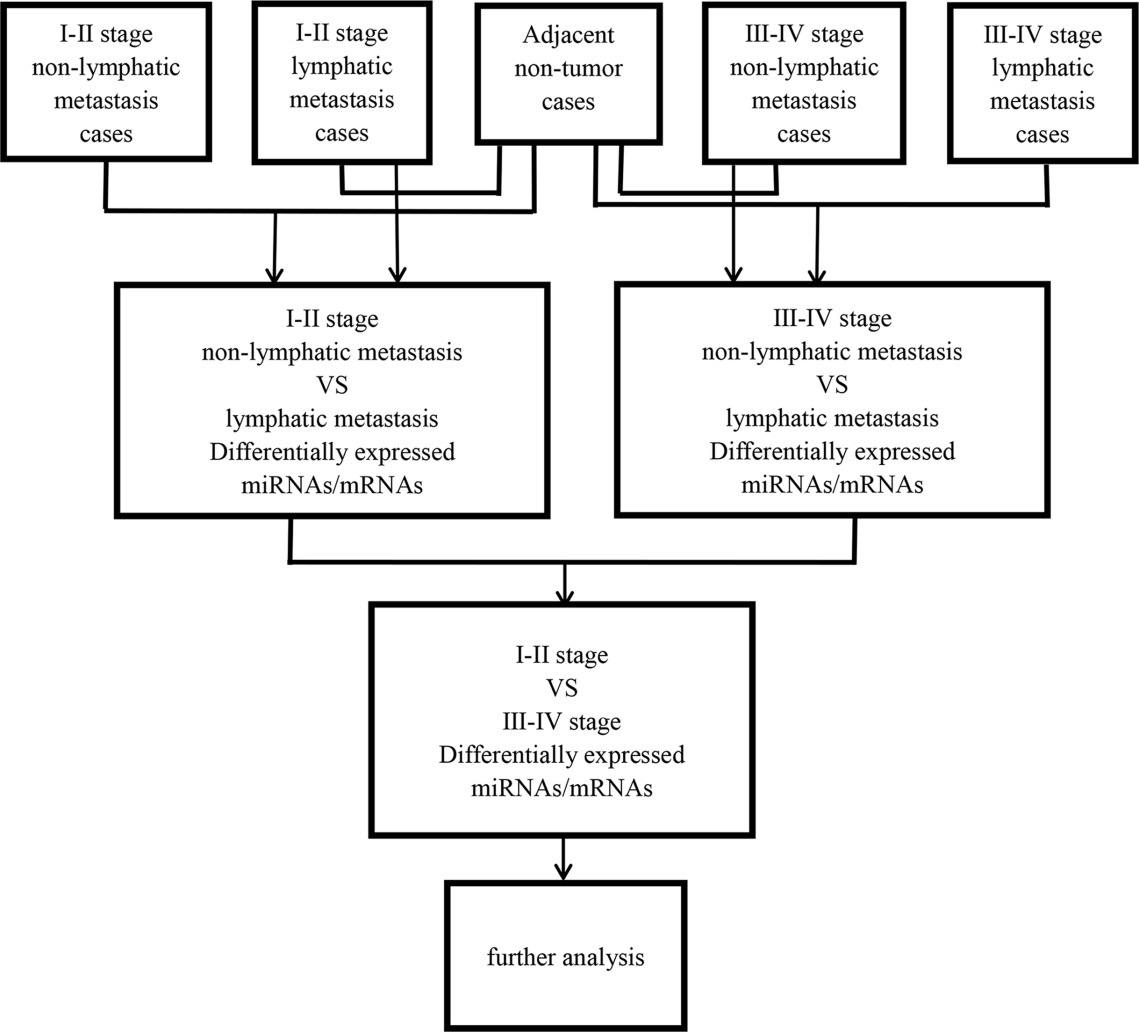
Flow chart for bioinformatics analysis.

Then, abnormally expressed miRNAs were compared in level 3 (fold changes >2, *p* < 0.05), including LUSC tumor samples vs. adjacent non-tumorous lung samples, lymphatic metastases of LUSC samples vs. non-lymphatic metastases of LUSC samples, and I–II stage vs. III–IV stage, respectively. Figure 1 shows the flow chart for bioinformatics analysis.

### Correlation analysis between LUSC-specific intersection miRNAs, characteristics of patients and overall survival

To explore the connection between LUSC-specific intersection miRNAs and clinical information, related miRNAs were found, using the comparative analysis of LUSC miRNA sequencing data in TCGA. The characteristics of patients was divided into five parts including gender, tumor grade, TNM stage, lymph node metastasis, and patient outcome assessment. Subsequently, Student *t*-test was used to evaluate the association between the LUSC-specific miRNAs and personal information using IBM SPSS Statistics Version 21.

Furthermore, to correlate specific intersection miRNAs with patients’ prognosis characteristics, the univariate Cox proportional hazards regression model and Mantel–Haenszel test were used to explore the association between specific intersection miRNAs and LUSC patient survival. At the same time, the overall survival (OS) curves were evaluated. LUSC-specific intersection miRNAs associated with OS were defined two independent subclasses by using hazard ratios (HRs), whose cutoff was that a positive signature showed HR < 1 and hazardous signature had HR > 1.

### Total RNA extraction and qRT-PCR verification

Total RNA was extracted tissue samples using TRIzol reagent (Invitrogen, Carlsbad, CA, USA) according to the instructions. Concentration and integrity of all extracted RNA were evaluated to be satisfactory.

To confirm the reliability and validity of TCGA data, we randomly selected five specific key miRNAs (miR-205-5p, miR-30a-3p, miR-30a-5p, miR-30c-2-3p and miR-30d-5p) of above intersection miRNAs. Then their actual expression levels in 47 pairs of LUSC samples were determined by quantitative real-time polymerase chain reaction (qRT-PCR).

A two-step protocol of reverse transcription reactions: First, 1 μg of RNA samples were pre-denatured (5 min at 65 °C and held at 4 °C). Then the 9 μl mixture including 2 μl 5 × RT buffer, 0.5 μl RT Enzyme Mix, 0.5 μl miRNA-specific stem-loop RT primers and 6 μl RNase-free water were added in the 1 μg pre-denatured RNA (37 °C × 15 min, 98 °C × 5 min and subsequently held at 4 °C).

Real-time PCR was then performed by Thunderbird SYBR qPCR Mix (QPS-201; TOYOBO, Osaka, Japan) according to the manufacturer’s protocol. The PCR reaction components comprised 1 μl cDNA, 5 μl Thunderbird SYBR qPCR Mix, 0.3 μl PCR primers (RiboBio, Guangzhou, China) and 3.4 μl RNase-free water. Then a two-step protocol [95 °C × 1 min; 40 cycles of (95 °C × 15 s, 60 °C × 30 s, 72 °C × 30 s)] was undertaken in on StepOnePlus PCR System (Applied Biosystems, Waltham, MA, USA). Results were normalized to the expression of U6. The results were expressed as mean ± SD. Each sample expression was calculated by the 2^−ΔΔCt^ method (ΔCt = Ct_miRNAs_ − Ct_U6_ and ΔΔCt = ΔCt_tumor tissues_ − ΔCt_adjacent non-tumor tissues_). Paired *t*-test was applied to for comparison between tumor tissues and the adjacent non-tumor lung tissues. In all cases, differences with *p* < 0.05 were considered to be statistically significant.

### ROC curve analysis of specific key miRNAs

According to the outcome of 332 patients, receiver operating characteristic (ROC) curve was also applied to evaluate the diagnosis value of the specific key miRNAs of LUSC.

### The prediction of microRNA target genes

It is known that mRNAs are regulated by corresponding miRNAs. So, to find the differential display of targeted mRNAs, samples were quartered similarly as abnormally expressed miRNAs, whose flow chart of bioinformatics analysis is presented in Fig. 1. In addition, interaction mRNA of LUSC-specific intersection miRNAs was predicted by using the Targetscan and the miRbase (Hsu et al., 2014). Last, according to the results of previous two steps, the mRNAs of the microRNA target genes were obtained.

### Differential expression of key mRNA function

To understand the potential biological processes and pathways of aberrant expression of intersection mRNAs, bioinformatics resources from database for annotation, visualization, and integrated discovery (DAVID) were used. We were only interested in the significant level (the enrichment score >2 and *p* < 0.05) of gene ontology (GO) biological processes and Kyoto Encyclopedia of Genes and Genomes (KEGG) pathways to analyze the potential role of these key mRNAs. Moreover, to further explore the relationship and function of intersection mRNAs, the PPI network was analyzed using the protein–protein interaction (PPI) network via STRING (http://string-db.org; Version 10.5).

## Results

### Significantly specific miRNAs in LUSC

In this study, 332 LUSC patients were selected from TCGA database. Then the expression of 332 LUSC and adjacent normal tissue miRNAs were compared to identify significantly differential miRNAs through a certain criterion (fold change ≥2 or ≤0.5, and *p* < 0.05) (Romero-Cordoba et al., 2012; Xu et al., 2014; Zhu et al., 2015).

**Figure 2:**
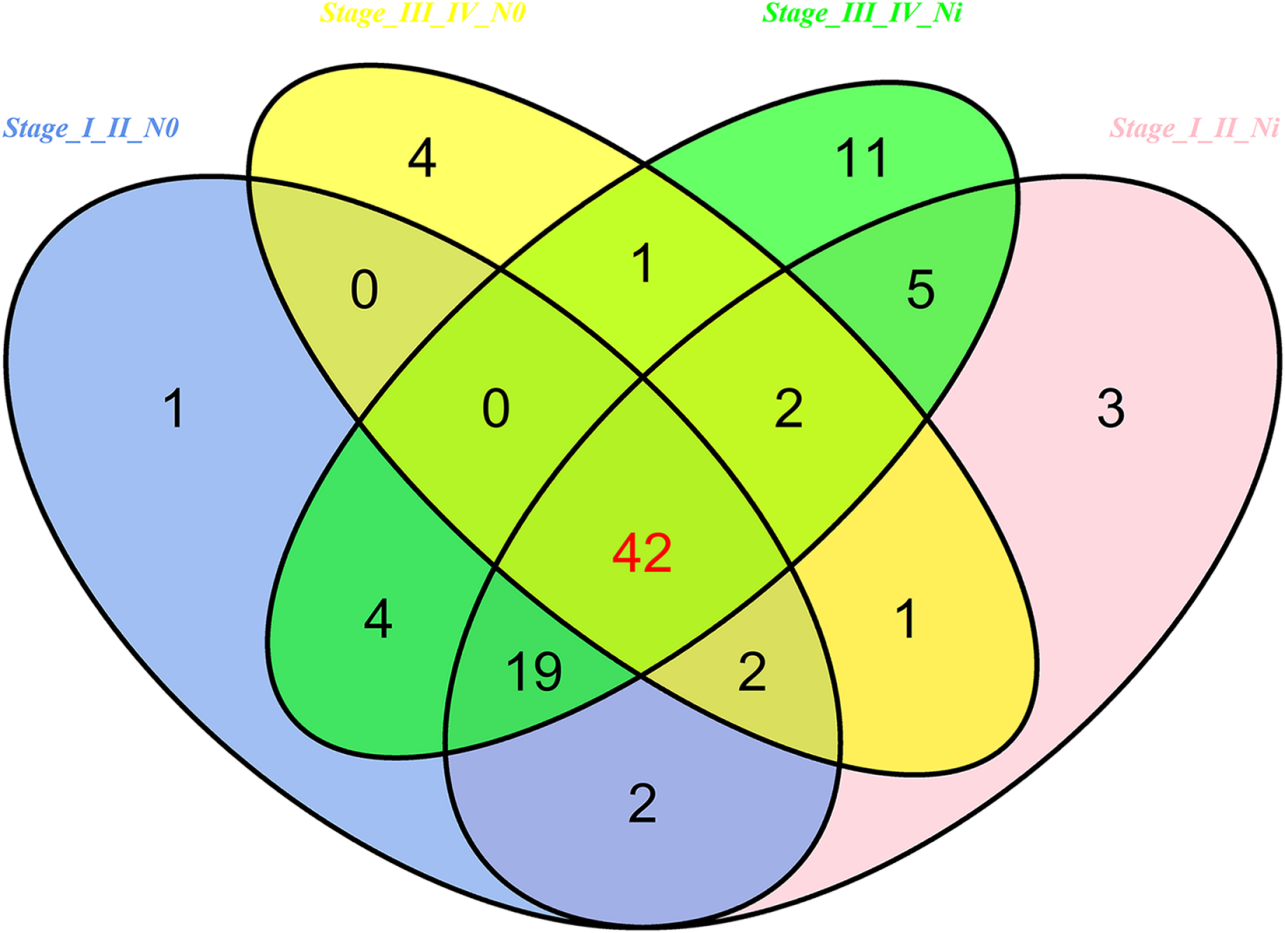
Venn diagram analysis of aberrantly expressed miRNAs between I–II Lymph/N, I–II non-Lym/N, III–IV Lymph/N and III–IV non-Lym/N. Lymph, lymphatic metastasis; non-Lym, non-lymphatic metastasis; N represents adjacent non-tumor lung tissues.

**Figure 3:**
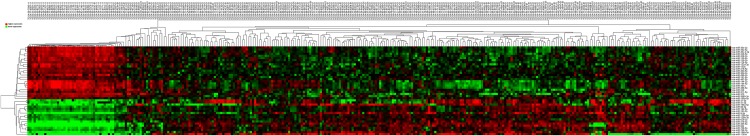
Cluster analysis of consistently differential miRNA expression. Red indicates that the miRNA has higher expression level; green indicates that the miRNA has lower expression. The samples at the end of “01” are cancerous tissues and the samples at the end of “11” are adjacent non-tumorous tissues.

Then 97 LUSC-associated abnormally expressed miRNAs were identified between 293 LUSC tumor tissue samples and 39 adjacent non-tumor tissues. Further, these 97 miRNAs between tumor stage and lymphatic metastasis were analyzed. And aberrantly expressed miRNAs were selected from four groups: (1) I–II stage (non-lymphatic metastases) LUSC tissues and adjacent non-tumorous lung tissue; (2) III–IV stage (non-lymphatic metastases) LUSC tissues and adjacent non-tumorous lung tissue; (3) I–II stage (lymph node metastasis) LUSC tissues and adjacent non-tumorous lung tissues; and (4) III–IV stage (lymph node metastasis) LUSC tissues and adjacent non-tumorous lung tissues. At last, we found 70, 52, 76 and 84 aberrantly expressed miRNAs in these four groups respectively (fold change >2, *p* < 0.05). To further enhance data reliability for bioinformatics analysis, 42 aberrantly expressed miRNAs in the intersection of these four groups were selected. Among these 42 intersection miRNAs, 17 miRNAs were upregulated and 25 miRNAs were downregulated (Figs. 2 and 3; Table 1).

**Table 1:** Abnormal expression of intersection miRNAs in LUSC.

miRNA	Regulation	miRNA_sequencing	Fold-change*
hsa-miR-144-5p	Down	GGAUAUCAUCAUAUACUGUAAG	0.085
hsa-miR-486-5p	Down	UCCUGUACUGAGCUGCCCCGAG	0.09
hsa-miR-144-3p	Down	UACAGUAUAGAUGAUGUACU	0.096
hsa-miR-338-5p	Down	AACAAUAUCCUGGUGCUGAGUG	0.1
hsa-miR-133a-3p	Down	UUUGGUCCCCUUCAACCAGCUG	0.11
hsa-miR-451a	Down	AAACCGUUACCAUUACUGAGUU	0.11
hsa-miR-139-3p	Down	UGGAGACGCGGCCCUGUUGGAGU	0.13
hsa-miR-30c-2-3p	Down	CUGGGAGAAGGCUGUUUACUCU	0.14
hsa-miR-139-5p	Down	UCUACAGUGCACGUGUCUCCAGU	0.15
hsa-miR-326	Down	CCUCUGGGCCCUUCCUCCAG	0.15
hsa-miR-30a-3p	Down	CUUUCAGUCGGAUGUUUGCAGC	0.18
hsa-miR-30a-5p	Down	UGUAAACAUCCUCGACUGGAAG	0.18
hsa-miR-375	Down	UUUGUUCGUUCGGCUCGCGUGA	0.18
hsa-miR-133b	Down	UUUGGUCCCCUUCAACCAGCUA	0.19
hsa-miR-338-3p	Down	UCCAGCAUCAGUGAUUUUGUUG	0.19
hsa-miR-3065-3p	Down	UCAGCACCAGGAUAUUGUUGGAG	0.2
hsa-miR-218-5p	Down	UUGUGCUUGAUCUAACCAUGU	0.24
hsa-miR-490-3p	Down	CAACCUGGAGGACUCCAUGCUG	0.25
hsa-miR-126-5p	Down	CAUUAUUACUUUUGGUACGCG	0.26
hsa-miR-30d-3p	Down	CUUUCAGUCAGAUGUUUGCUGC	0.26
hsa-miR-511-5p	Down	GUGUCUUUUGCUCUGCAGUCA	0.26
hsa-miR-101-5p	Down	CAGUUAUCACAGUGCUGAUGCU	0.28
hsa-miR-190a-5p	Down	UGAUAUGUUUGAUAUAUUAGGU	0.28
hsa-miR-30d-5p	Down	UGUAAACAUCCCCGACUGGAAG	0.28
hsa-miR-497-5p	Down	CAGCAGCACACUGUGGUUUGU	0.3
hsa-miR-629-3p	Up	GUUCUCCCAACGUAAGCCCAGC	3.58
hsa-miR-130b-3p	Up	CAGUGCAAUGAUGAAAGGGCAU	3.77
hsa-miR-130b-5p	Up	ACUCUUUCCCUGUUGCACUAC	3.94
hsa-miR-96-5p	Up	UUUGGCACUAGCACAUUUUUGCU	4.82
hsa-miR-182-5p	Up	UUUGGCAAUGGUAGAACUCACACU	4.9
hsa-miR-708-3p	Up	CAACUAGACUGUGAGCUUCUAG	5.9
hsa-miR-31-3p	Up	UGCUAUGCCAACAUAUUGCCAU	6.18
hsa-miR-183-5p	Up	UAUGGCACUGGUAGAAUUCACU	7.21
hsa-miR-31-5p	Up	AGGCAAGAUGCUGGCAUAGCU	8.48
hsa-miR-708-5p	Up	AAGGAGCUUACAAUCUAGCUGGG	8.96
hsa-miR-196a-5p	Up	UAGGUAGUUUCAUGUUGUUGGG	15.61
hsa-miR-210-3p	Up	CUGUGCGUGUGACAGCGGCUGA	19.68
hsa-miR-196b-5p	Up	UAGGUAGUUUCCUGUUGUUGGG	19.72
hsa-miR-9-5p	Up	UCUUUGGUUAUCUAGCUGUAUGA	24.89
hsa-miR-944	Up	AAAUUAUUGUACAUCGGAUGAG	50.7
hsa-miR-1269a	Up	CUGGACUGAGCCGUGCUACUGG	65.09
hsa-miR-205-5p	Up	UCCUUCAUUCCACCGGAGUCUG	143.65

**Note:**

* These miRNAs have significantly different expression (fold change ≥2 or ≤0.5, and *p* < 0.01).

### Association between LUSC-specific miRNAs and characteristics of patients

After analyzing the correlation between expression of the 42 LUSC-specific intersection miRNAs and their related characteristics of patients (race, gender, age, TNM stage, lymphatic metastases, and patient outcome status in the TCGA database), six specific miRNAs were significantly abnormal in clinical information (*p* < 0.05, Table 2).

**Table 2:** The correlations between LUSC-specific intersection miRNAs and characteristics of patients.

Comparisons	Downregulated	Upregulated
Gender (female vs. male)	hsa-miR-629-3p	hsa-miR-511-5p
Lymphatic metastasis (no vs. yes)	hsa-miR-130b-5p	hsa-miR-30c-2-3p
	hsa-miR-130b-3p	
	hsa-miR-629-3p	
Patient outcome assessment (dead vs. alive)		hsa-miR-30a-3p

Two miRNAs (hsa-miR-629-3p and hsa-miR-511-5p) were aberrantly expressed in gender, four miRNAs (hsa-miR-30c-2-3p, hsa-miR-130b-5p, hsa-miR-629-3p and hsa-miR-130b-3p) were aberrantly expressed in lymphatic metastasis and one miRNA (hsa-miR-30a-3p) were aberrantly expressed in patient outcome assessment, however, none of aberrantly expressed miRNA was discovered in race, age, TNM stage and tumor stage.

### Survival analysis

**Figure 4:**
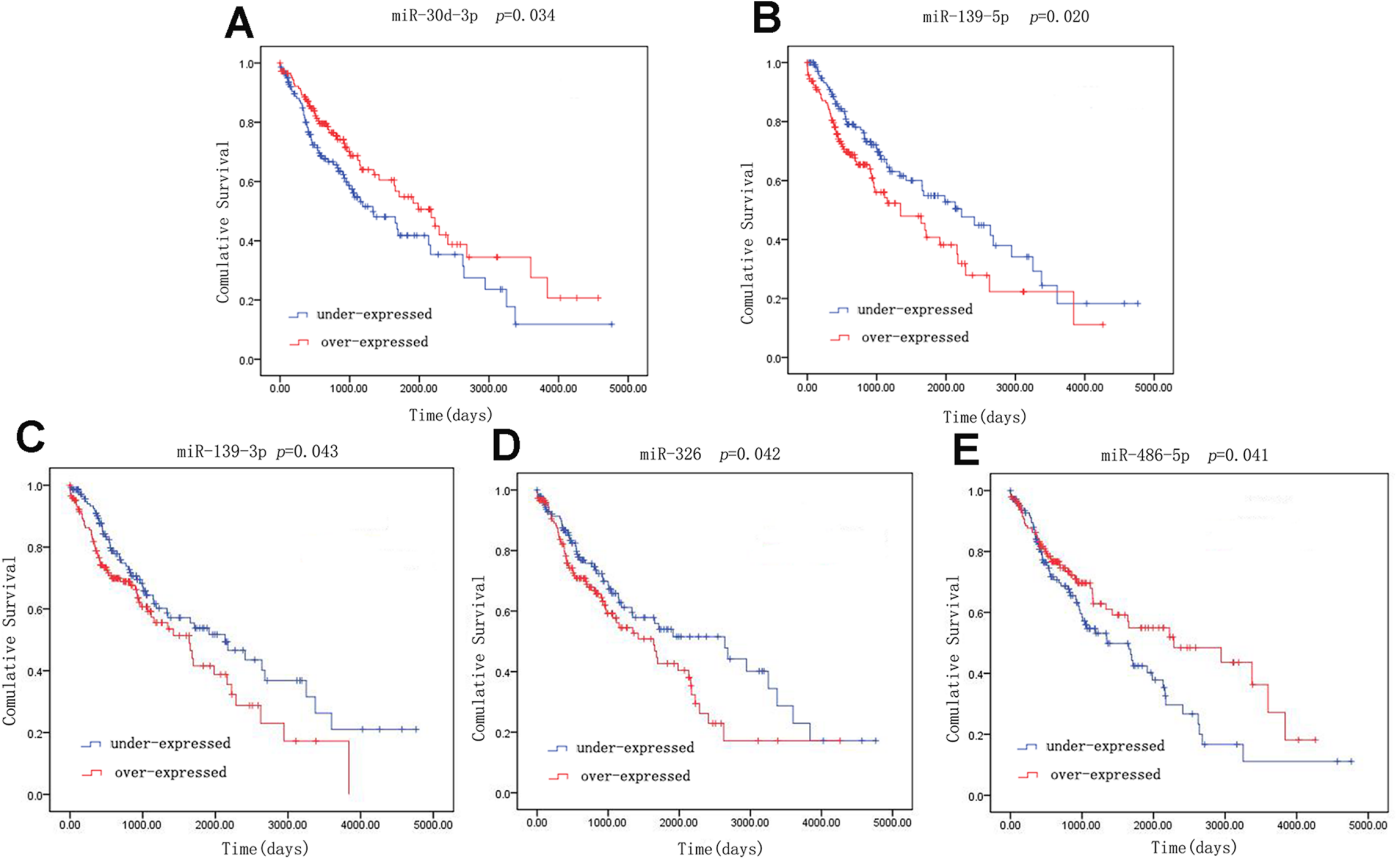
Kaplan–Meier survival curves for five miRNAs associated with overall survival. Horizontal axis: overall survival time, days; Vertical axis: survival function.

A univariate Cox model was used to identify the 42 LUSC-specific intersection miRNAs correlated with OS. Five of these miRNAs were significantly associated with the overall survival status of 332 LUSC patients (log-rank *p* < 0.05): three (miR-139-5p, miR-139-3p and miR-326) negatively (*p* < 0.05) and two (miR-30d-3p and miR-486-5p) positively (*p* < 0.05) (Fig. 4).

### qRT-PCR verification

**Figure 5:**
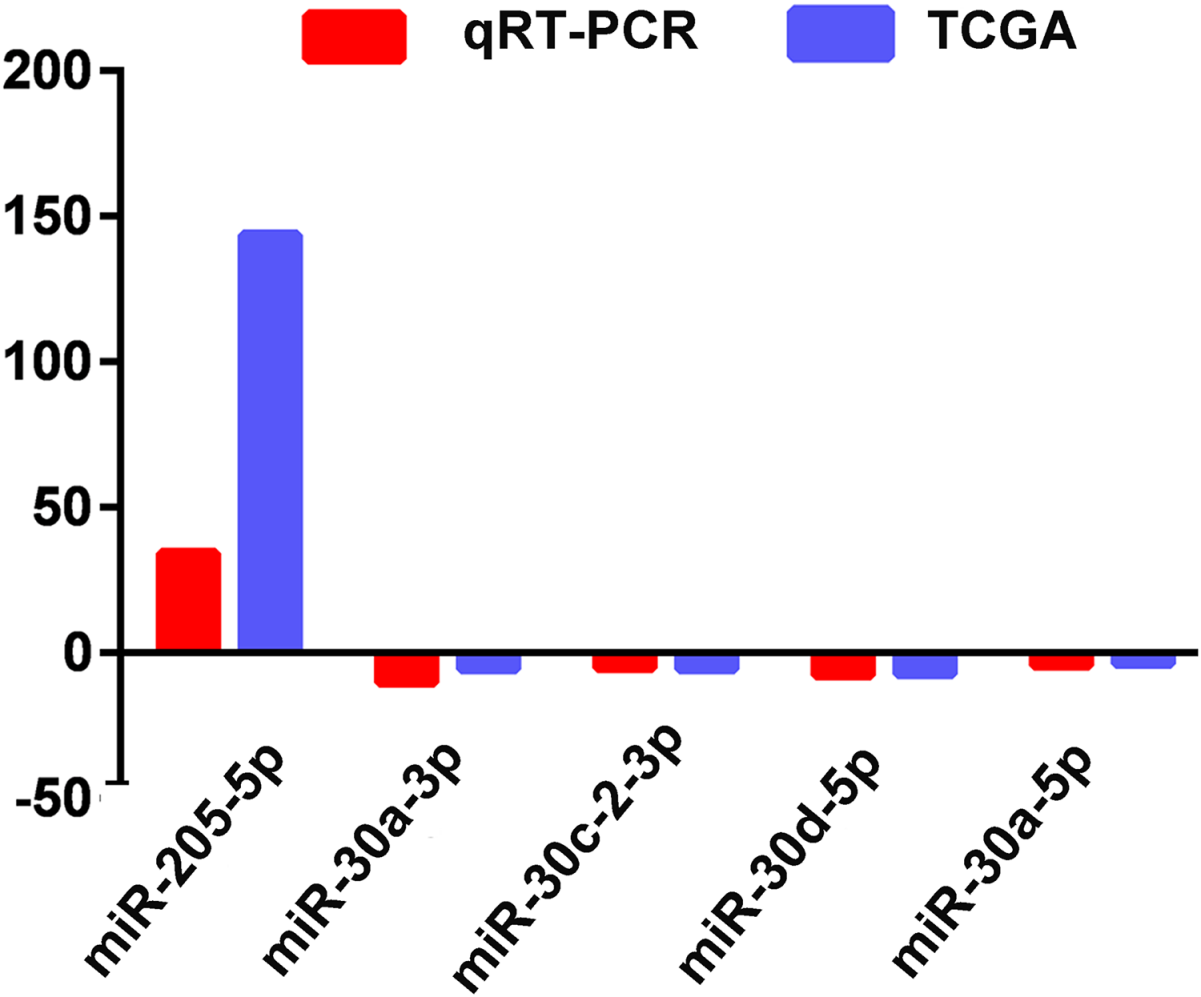
Quantitative real-time polymerase chain reaction validation of five aberrantly expressed key miRNAs. Comparison of fold change (2-Ct) of miRNAs between TCGA and qRT-PCR results.

The actual expression levels of five specific key miRNAs (miR-205-5p, miR-30a-3p, miR-30a-5p, miR-30c-2-3p and miR-30d-5p) were measured by qRT-PCR. The results demonstrated that miR-205-5p was upregulated in LUSC tumor tissues, while miR-30a-3p, miR-30a-5p, miR-30c-2-3p and miR-30d-5p were significantly downregulated in LUSC tumor tissues. Expression levels of these five miRNAs were consistent with the TCGA data (Fig. 5; Table 1).

### ROC curve analysis of specific key miRNAs

**Figure 6:**
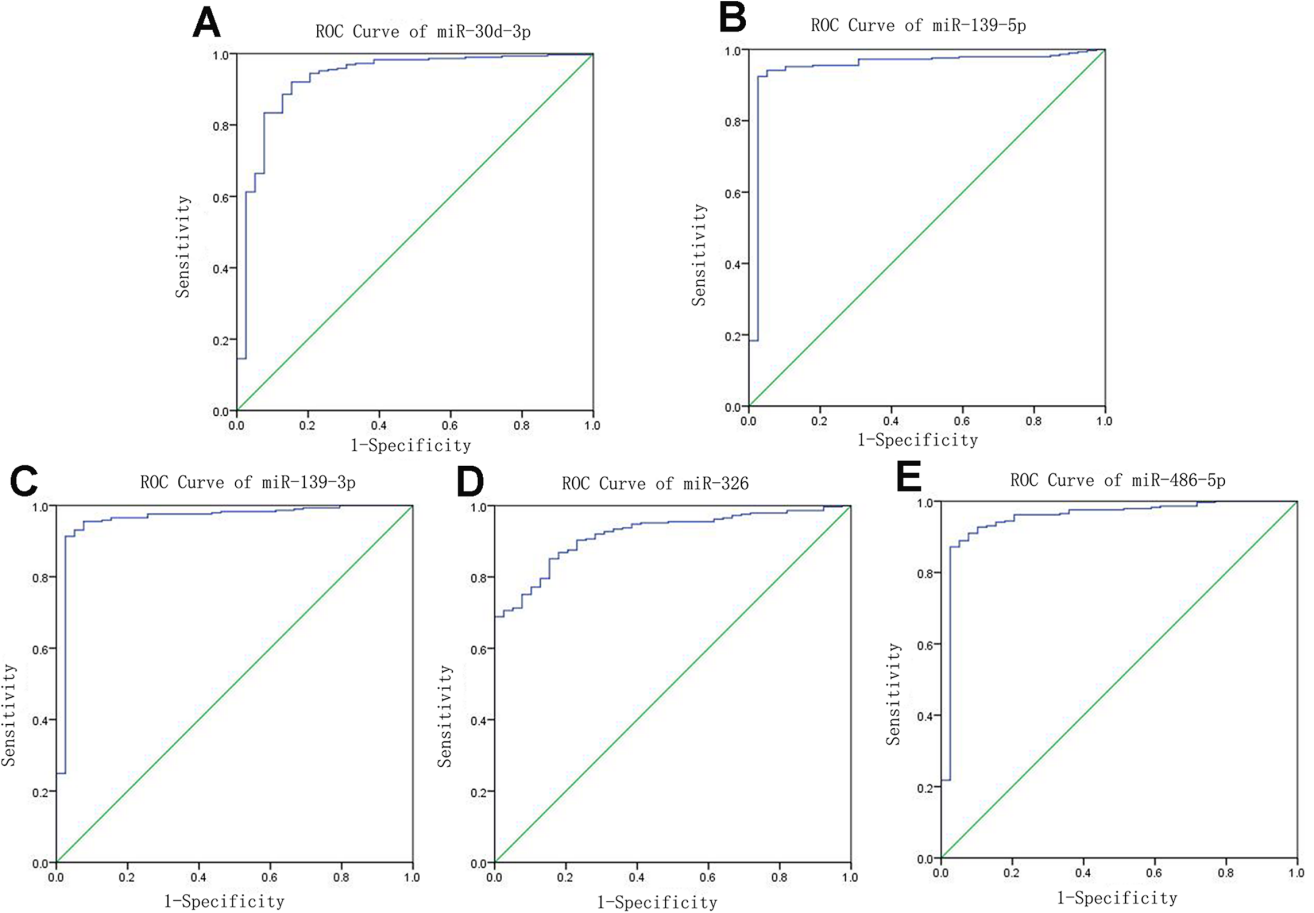
Receiver operating characteristic curve analysis of specific key miRNAs.

Receiver operating characteristic curves were also used to assess diagnosis value of the LUSC-specific intersection miRNAs which related with overall survival. The area under ROC curve (AUC) = 0.930, 0.950, 0.961, 0.919 and 0.952 for miR-30d-3p, miR-139-5p, miR-139-3p, miR-326 and miR-486-5p (*p* < 0.01, Fig. 6), which are the score all higher the cutoff (0.7) and could be considerable biomarkers for early diagnosis of LUSC.

### Prediction of target genes

**Figure 7:**
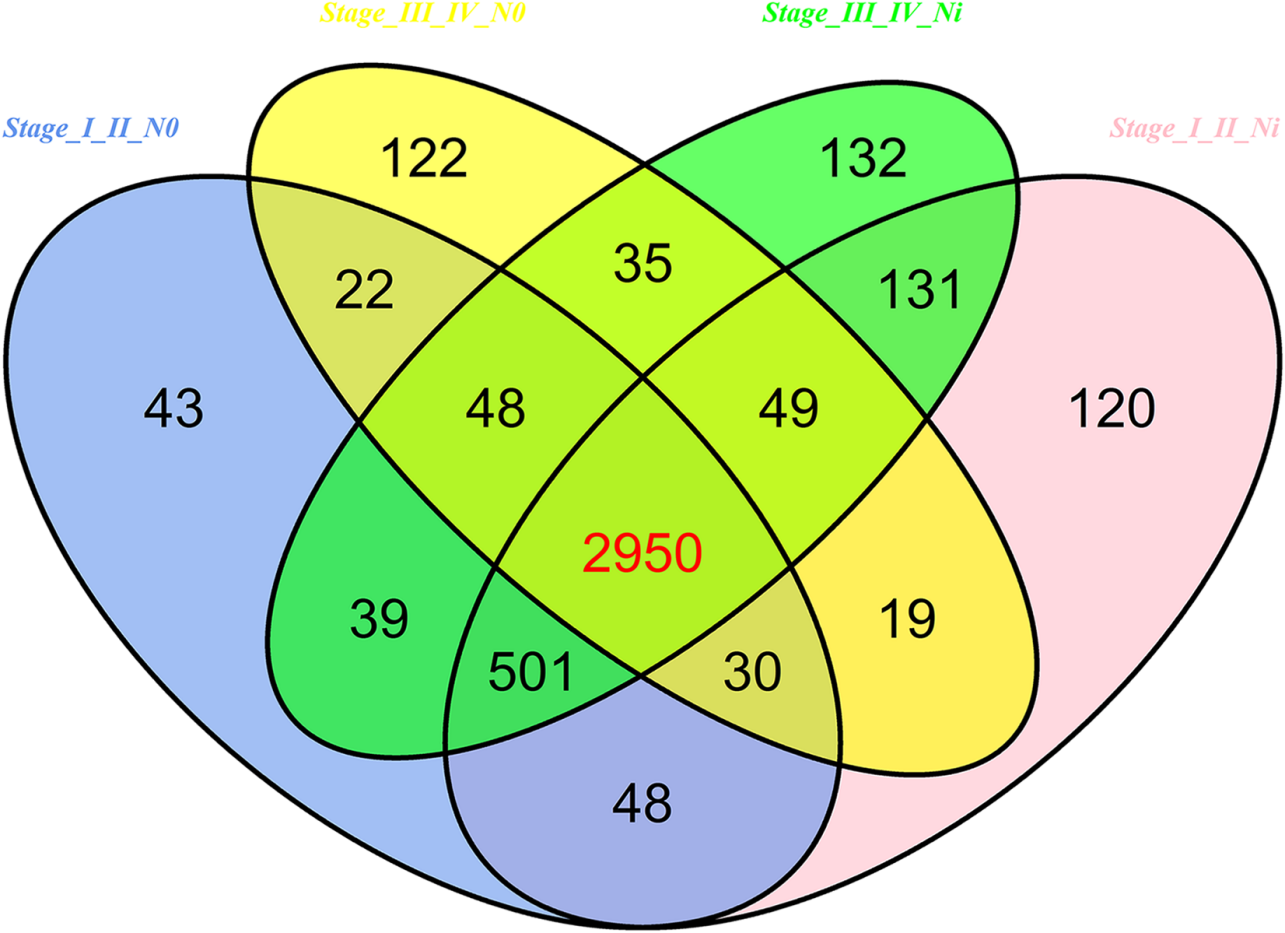
Venn diagram analysis of aberrantly expressed mRNAs between I–II Lymph/N, I–II non-Lym/N, III–IV Lymph/N and III–IV non-Lym/N. Lymph, lymphatic metastasis; non-Lym, non-lymphatic metastasis; N represents adjacent non-tumor lung tissues.

In 474 LUSC samples from TCGA database, 18,633 mRNAs were obtained from the TCGA database. According to the cutoffs that was fold change ≥2 or ≤0.5, and *p* < 0.05, significantly aberrantly expressed mRNAs were identified between 293 LUSC tumor tissue samples and 39 adjacent non-tumor tissues. In total, 14,689 LUSC-associated abnormally expressed mRNAs were selected to be further compared between tumor stage and lymphatic metastasis. 3,681 aberrantly expressed mRNAs were selected from comparisons of I–II stage (non-lymph node metastasis) LUSC specimens and adjacent non-cancerous lung tissues, 3,848 from comparisons of I–II stage (lymphatic metastasis) with adjacent non-cancerous lung tissues, 3,275 from comparisons of III–IV stage (non-lymph node metastasis) and adjacent non-cancerous tissues, and 3,885 from comparisons of III–IV stage (lymphatic metastasis) and adjacent non-cancerous tissues. As a result, we selected 2,950 aberrantly expressed mRNAs (1,770 downregulated and 1,180 upregulated) from the intersection of the above four groups (Fig. 7).

The next step was to predict the mRNA of LUSC-specific intersection miRNAs. 577 interaction mRNAs were obtained by using the Targetscan and the miRbase.

Based on the above steps, the interaction mRNAs that appeared simultaneously in previous results were selected. It is shown that 2,950 aberrantly expressed mRNAs in the first step contained all 577 interaction mRNAs in the second step, which meant that the 577 mRNAs were the target genes.

### Function analysis

Furthermore, functions of LUSC-specific intersection miRNAs were predicted by interaction mRNAs with DAVID bioinformation.

**Figure 8:**
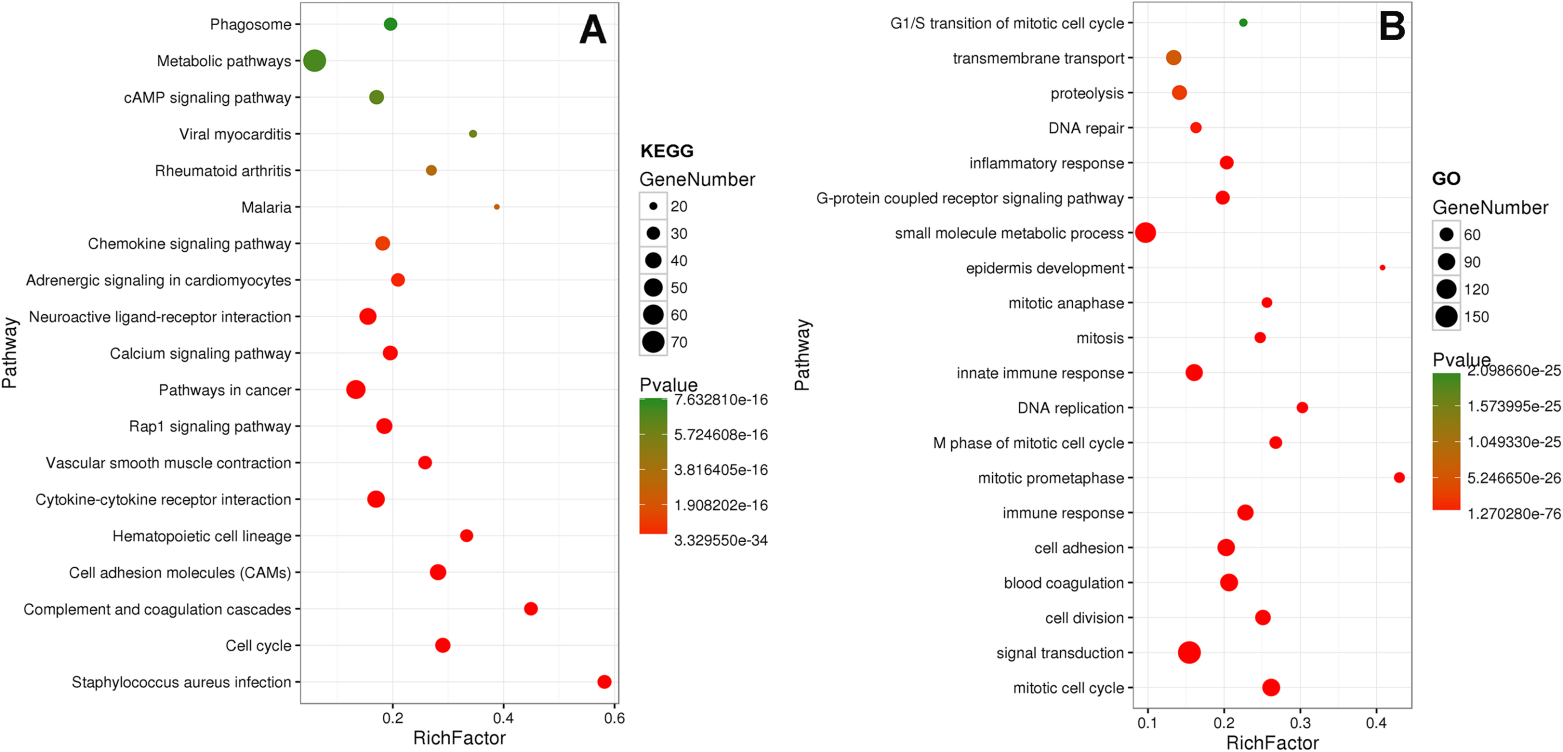
KEGG and GO analysis of related genes.

**Figure 9:**
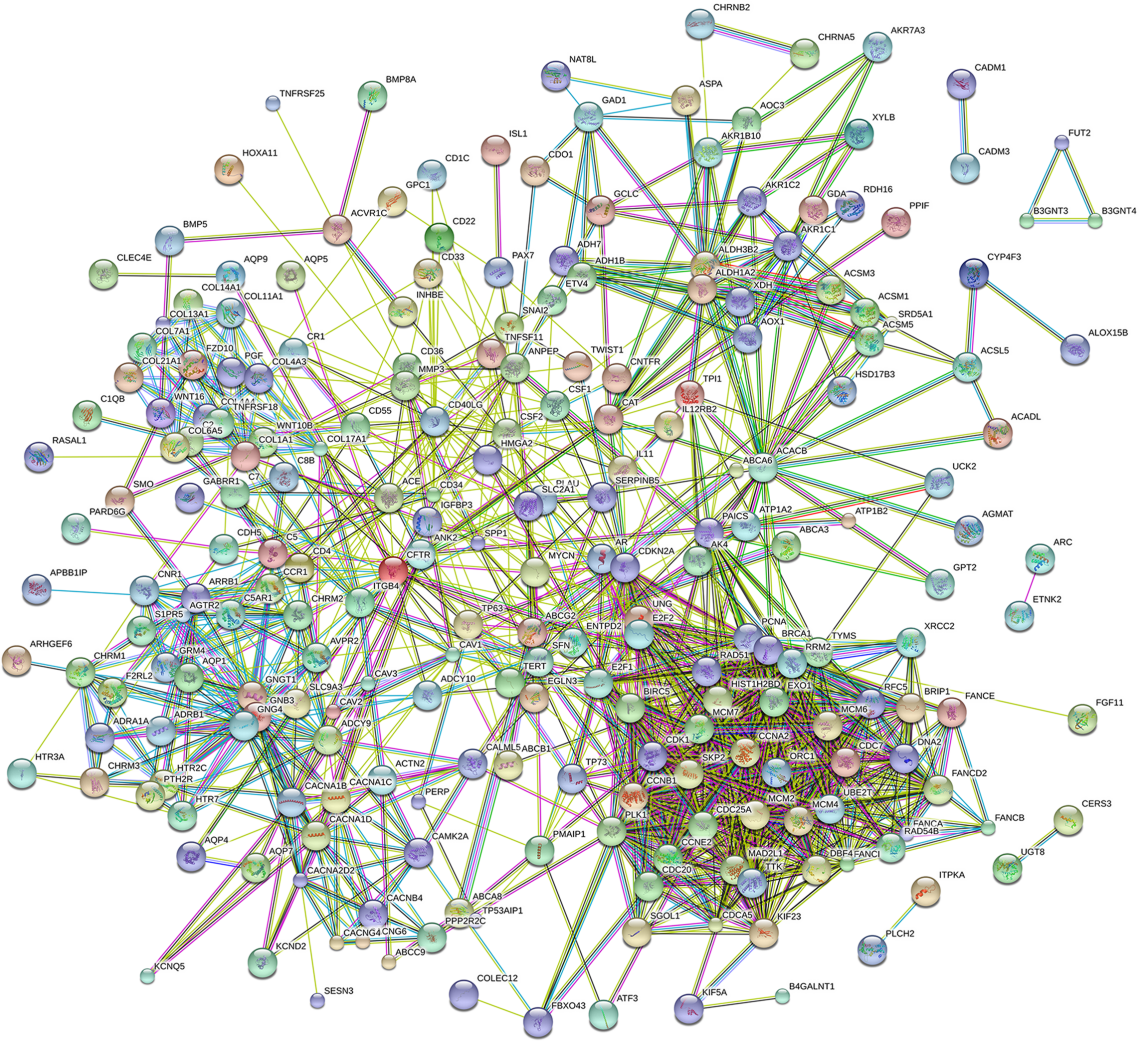
Protein–protein interaction network of 577 mRNAs.

Eighty-one pathways were indicated by KEGG pathway analysis, and 433 GO terms (*p* < 0.05 and enrichment >2) were identified by analyzing the enrichment of these genes. Moreover, KEGG pathway analysis showed the most significant networks were *Staphylococcus aureus* infection (path ID: 05150) and cell cycle (path ID: 04110) (Fig. 8). The result suggested that five of the top 20 pathways were tumorous pathways including cell cycle, cell adhesion molecules (CAMs), cytokine–cytokine receptor interaction, Rap1 signaling pathway, and pathways in cancer. There were other tumor-related pathways such as cAMP signaling pathway, metabolic pathways, phagosome, PI3K-Akt signaling pathway, miRNAs in cancer, small cell lung cancer, and hippo signaling pathway in the other significantly differentially altered pathways. The highest enriched GO terms were mitotic cell cycle (GO: 0000278) and GO 0007165 (GO: 0007268) (Fig. 8). The relationship and function of 577 mRNAs were revealed in the PPI network (Fig. 9).

## Discussion

Lung squamous cell carcinoma is one of the most common subtypes of lung cancer, remaining large cancer-related death in China (Peng et al., 2015). It is reported that lung cancer may be caused by many factors, including environment (smoky coal, radon) (Kim et al., 2014; Torres-Duran et al., 2015), life style (smoking, diets) (Gnagnarella et al., 2013; Yang, 2011), human papillomavirus (Hsu et al., 2013), chronic pulmonary infection (it can be caused by *S. aureus*) (Mulanovich, Mulanovich & Rolston, 2008; Parker & Prince, 2012), genetic factors (family history) (Zhou et al., 2014) and so on, which may be the risk factors of LUSC. The development of individualized accurate diagnosis and therapy have been faced with huge challenges due to immense difference in multiple parameters such as molecular, pathology, surgery, and radiology among LUSC patients (Pavel & Vasile, 2016). Diagnosis, medical treatment, surgery and prognosis of LUSC have been drawn much attention and mushroom growth, but the five-year overall survival is still unsatisfactory (Liu, Chen & Yang, 2017). According to past decade reports, miRNAs can serve as potential non-invasive biomarkers for cancer, more effective and accurate (Lin et al., 2014; Su et al., 2012; Yoshino et al., 2013; Youssef et al., 2011). There is growing evidence that miRNAs play a key role in the development and progression of lung cancer, as crucial biomarkers for early diagnosis, pathological classification, clinical treatment and prediction of outcome for lung cancer (Melkamu et al., 2010; Tian et al., 2016).

In this study, aberrantly expressed miRNAs in LUSC were identified from the TCGA database. Based on miRNA sequencing profiles in TCGA, we explored the relationship between LUSC miRNAs and different characteristics of patients (gender, tumor grade, TNM staging system, lymph node metastasis, and patient outcome assessment). The association between LUSC miRNAs and overall survival was also analyzed by OS curves. Then the target genes of abnormally expressed miRNAs were selected by related analysis. At last, we further predicted gene function and biological pathway, analyzed the protein–protein interaction.

After abnormally expressed miRNAs from GCTA database compared in level 3, 42 aberrantly expressed miRNAs were obtained, including 17 upregulated and 25 downregulated miRNAs. Among them, some have been observed in LUSC. Tan et al. (2011) reported that downregulation of hsa-miR-486-5p could distinguish LUSC from other lung cancers. Jiang et al. (2013) also reported that expressions of miR-205-5p in LUSC were significantly higher than in lung adenocarcinoma samples, which could be beneficial to the precise diagnosis. These results showed that their findings were consistent with the findings of the present study. However, a few of these abnormally expressed miRNAs have not been verified. We wish that the best expectation is to establish 100% concordance between the conventional diagnosis and miRNA-based methods.

Through exploring the associations between the 42 LUSC-specific key miRNAs and characteristics of patients (race, gender, age, TNM stage, lymphatic metastasis, and patient outcome), six miRNAs were found to be related to clinical information, including gender, lymphatic metastasis, and patient outcome assessment. Among them hsa-miR-511-5p has not been reported in any cancer. Zhang et al. (2017) found that downregulation of miR-30c-2-3p and miR-30a-3p could increase the risk of lung cancer using conditional logistic regression analysis, serving as non-invasive biomarkers for lung cancer diagnosis (Cazzoli et al., 2013; Jin et al., 2017). Wang et al. (2017) reported that miR-629-3p might serve as a new biomarker and potential therapeutic target for lung metastases of breast cancer, which was in accord with the association between miR-629-3p and lymphatic metastasis in the present study. There is no study on miR-130b-5p in LUSC but in breast cancer and ovarian cancer (Chang et al., 2015; Wang et al., 2014). Mitra et al. (2014) claimed that miR-130b-3p could be the biomarker in non-small cell lung cancer by the network consisting of miRNAs, transcription factors and predicted target genes. The results in the present study and related reports support the signature of six miRNAs in predicting LUSC.

To explore the associations between 42 LUSC-specific key miRNAs and patients’ survival were analyzed, five miRNAs were found to be related to LUSC overall survival. Some related reports has reported that miR-139-5p (Hiroki et al., 2010; Yonemori et al., 2016), miR-139-3p (Liu et al., 2014; Yonemori et al., 2016), miR-326 (Wang et al., 2013), miR-486-5p (Madhavan et al., 2016) were related to overall survival in some cancers, but there is no study about miR-30d-3p.

To validate the analyzed results from TCGA data, 5 of the 42 LUSC-specific key miRNAs (miR-205-5p, miR-30a-3p, miR-30a-5p, miR-30c-2-3p and miR-30d-5p) were randomly selected and measured using qRT-PCR. The results indicated that TCGA analysis and qRT-PCR results from 47 LUSC patients were in 100% agreement.

Then ROC was also used to determine the five specific key miRNAs as the diagnosis value of LUSC detection. In the future, above 11 miRNAs will be the priority in the lung cancer studies, using lung cancer tissues and blood samples to examine the relationship between lung cancer and the environment to identify risk factors.

In order to further understand the function of LUSC-specific intersection miRNAs, their target genes were obtained. We analyzed the enrichment and pathways of these target mRNAs by DAVID. After related analysis, 577 mRNAs were obtained as the target genes and bioinformatic analyzed, revealing that some tumor-related genes were significant in cancers and protein–protein interaction. Similarly, many genes of them have reported on LUSC (Li et al., 2016; Schlensog et al., 2016; Sun et al., 2017).

Huang, Yang & Cai (2015) also used the TCGA to find microRNA let-7a and miR-31 as novel candidate key roles in LUSC, however, the present study not only found key miRNAs by larger samples and more analytical methods, but also verified the reliability of TCGA findings by small sample. The greatest merit of the study is that it opens up a new method to predict and identify related miRNAs of LUSC. It is based on large-scale public data platform, reliable and representative. The findings of TCGA can play a guiding role for LUSC diagnosis and prognosis assessment. If the new biomarkers of LUSC is further confirmed, these miRNAs will be applied to the clinic for non-invasive diagnosis.

Nevertheless, a few limitations may exist in the present study. First, TCGA LUSC dataset had relatively high censored rate, having the influence on related analysis. Second, the present study only used small sample to verify. The diagnostic value of these miRNA biomarkers should be validated by a large number of proof tests or in the independent long-term cohort.

## Conclusion

In conclusion, the present study identified LUSC-specific miRNAs as potential diagnosis biomarkers for LUSC patients. These LUSC-specific miRNAs can be further validated using independent large-sample-size cohorts, and future functional studies are necessary to explore the underlying mechanisms of these LUSC-specific miRNAs.

## Supplemental Information

10.7717/peerj.4101/supp-1 Supplemental Information 1 Clinical features of patients.

10.7717/peerj.4101/supp-2 Supplemental Information 2 Raw data of miRseq.

## Additional Information and Declarations

### Data Availability

The following information was supplied regarding data availability: The LUSC RNA expression data (level 3) of patients were collected from the TCGA Data Portal, including miRNA (provided as Supplemental Dataset Files) and mRNA expression (Yang, Sheng (2017): LUSC.mRNAseq.txt. figshare. https://doi.org/10.6084/m9.figshare.5547847).
